# Practical Barriers to Medication Adherence: What Do Current Self- or Observer-Reported Instruments Assess?

**DOI:** 10.3389/fphar.2020.00572

**Published:** 2020-05-13

**Authors:** Amy Hai Yan Chan, Vanessa Cooper, Helen Lycett, Rob Horne

**Affiliations:** ^1^Centre of Behavioural Medicine, Department of Practice and Policy, University College London, London, United Kingdom; ^2^School of Pharmacy, Faculty of Medical and Health Sciences, University of Auckland, Auckland, New Zealand; ^3^Spoonful of Sugar Ltd, UCL-Business Spin-out Company, London, United Kingdom

**Keywords:** adherence–compliance–persistence, medication, measurement, self-report measures, practical factors, review (article), patient report, PRO (patient reported outcomes)

## Abstract

**Introduction:**

Practical adherence barriers (e.g., medication frequency) are generally more amenable to intervention than perceptual barriers (e.g., beliefs). Measures which assess adherence barriers exist, however these tend to measure a mix of factors. There is a need to identify what practical barriers are captured by current measures.

**Aim:**

To identify and synthesise the practical adherence barriers which are assessed by currently available self- or observer-report adherence measures.

**Methods:**

A search for systematic reviews of self- or observer-report report adherence measures was conducted. Three electronic databases (Embase, Ovid Medline, and PsycInfo) were searched using terms based on adherence, adherence barriers and measures. Systematic reviews reporting on adherence measures which included at least one self- or observer-report questionnaire or scale were included. Adherence measures were extracted and coded on whether they addressed perceptual or practical barriers, or both. Practical items were then analysed thematically.

**Results:**

Following screening of 272 initial abstracts, 20 full-text papers were reviewed. Four were excluded after full-text review, leaving 16 systematic reviews for data extraction. From these, 187 different adherence measures were extracted and coded, and 23 unique measures were identified as assessing practical barriers and included in the final analysis. Seven key themes were identified: formulation; instructions for use; issues with remembering; capability—knowledge and skills; financial; medication supply and social environment.

**Conclusion:**

Existing adherence measures capture a variety of practical barriers which can be grouped into seven categories. These findings may be used to inform the development of a measure of practical adherence barriers.

## Introduction

Poor adherence to medication in both long- and short-term conditions increases the risk of morbidity, mortality, and cost of care (Roebuck et al., 2011). Despite many years of research into non-adherence and the development of many different interventions to improve poor adherence, medication adherence remains largely an unresolved public health issue. In the European Union (EU) alone, non-adherence is estimated to cost the EU 1.25 billion each year from lost health gains and poor health outcomes (Pharmaceutical Group of the European Union, 2008). Part of the difficulty with addressing non-adherence is its complexity, as adherence can be influenced by multiple factors (Nieuwlaat et al., 2014). These factors can be enablers or barriers to adherence and broadly classified as either perceptual or practical, an approach which has been described in The National Institutes of Clinical Excellence (NICE) adherence guidelines (Horne et al., 2005; Nunes et al., 2009). Perceptual factors refer to factors which arise primarily from internal cognitive processes, such as motivation, emotions, or patient perceptions and beliefs of the illness and treatment, while practical factors refer primarily to external environmental factors relating to the individual, treatment, or society which can affect behaviour (Horne et al., 2019). Examples include the look and feel of the medication, how easy it is to access the medication, and the structure of the healthcare system. These perceptual and practical factors can lead to intentional non-adherence or unintentional non-adherence, respectively (Clifford et al., 2008).

An important first step when addressing any healthcare problem or behavior is to identify and assess the factors which are leading to the behavior, so that interventions can be designed to minimise or remove these factors. While no single type of adherence intervention has been shown to be more effective in improving adherence than others (Nieuwlaat et al., 2014), interventions that are tailored to address the specific adherence barriers faced by an individual are more likely to be effective than a one-size fits all approach (Kassavou and Sutton, 2017). In order to inform the development of a tailored intervention, there is a need for a measure which can (a) identify the types of factors influencing the behavior, and (b) quantify the degree that the various factors are influencing the behavior.

In recent years, there has been an increasing focus on the identification and assessment of perceptual factors influencing adherence, with the application of different theories and models to explain non-adherence (Holmes et al., 2014). The Beliefs about Medicines questionnaire is a valid and reliable measure for quantifying perceptual factors influencing adherence (Horne et al., 1999). A systematic review and meta-analysis showed that non-adherence to medication prescribed for a range of long-term conditions was predicted by doubts about the necessity for treatment (necessity beliefs) and concerns about adverse effects (concerns) (Horne et al., 2013). However, while these perceptual barriers are important factors influencing intentional non-adherence, they may not explain unintentional non-adherence. Even if a person intends to take their medication as prescribed, they may be prevented from doing so by limitations in capacity or resource (i.e., practical adherence barriers) such as deficiencies in memory or access to supply of medicines (Briesacher et al., 2007; Garfield et al., 2011). For example, forgetting has been cited as one of the most common reasons for non-adherence (Taylor et al., 2002; Martin et al., 2005; Shubber et al., 2016; Jamison et al., 2017). There has been a focus on perceptual factors but identifying practical factors are equally important in influencing adherence as perceptual and practical factors influence each other. Changing an individual’s practical barriers (e.g., by making a treatment easier to take) can lead to changes in perceptual barriers (e.g., by increasing motivation to take a treatment and vice versa (e.g., increases in perceived necessity of the treatment can translate into increased efforts in the individual to overcome any previous practical difficulties in taking the medication). Identifying and quantifying practical factors influencing non-adherence is thus an important first step in promoting adherence, as practical factors are often more easily amenable to changes in the physical environment compared to perceptual barriers.

There are currently several self- or observer-reported medication adherence measures available which identify factors which influence adherence (Garfield et al., 2011; Nguyen et al., 2014). These measures range from self- or observer-reported questionnaires to task-based tools which include instruments and surveys. The measures vary in terms of their purpose, what type of factors they measure (perceptual or practical), how they measure these factors (dichotomous or numerical scale), and whether or not the measure has been validated (Nguyen et al., 2014). Some questionnaires also measure adherence itself (i.e., the extent of adherence or medication-taking), as well as the factors influencing adherence (Nguyen et al., 2014). Few measures however distinguish between the different types of non-adherence (i.e., intentional versus unintentional non-adherence), nor measure both perceptual and practical adherence barriers (Garfield et al., 2011), limiting the ability of existing measures to be used to tailor interventions to individual needs.

The aim of this paper is to identify and synthesise the practical factors which are currently measured by self- or observer-reported adherence measures. There have been several studies reporting on adherence measures including systematic reviews of adherence measures; however, the large number of studies, and the variation in breadth and scope of the reviews, make it difficult for those involved in the design of adherence interventions and health policies to identify, extract, and interpret what practical factors are assessed by current adherence measures. As such a review of reviews was deemed necessary to identify what practical factors are currently assessed by adherence measures. Previous reviews of adherence measures or barriers have also not focused on practical barriers to adherence, or included self-report measures only (i.e., observer-rated instruments were excluded) or were limited to measures which have been correlated against a comparison measure of medication-taking behavior (Kardas et al., 2013; Nguyen et al., 2014). Furthermore, prior reviews have focused on synthesising measures which assess adherence *per se* (i.e., medication-taking), rather than measures which evaluate *factors* influencing adherence (Garfield et al., 2011).

This review of systematic reviews will identify and synthesise the different practical barriers which influence adherence that are currently included in self- or observer-report adherence measures.

## Methods

A search for systematic reviews of self- or observer-report adherence measures was conducted using Embase, Ovid Medline, and PsycInfo electronic databases. Search terms based on adherence, adherence barriers, and questionnaires or measures were used (see **Appendix 1** for full search strategy). The search was limited to systematic reviews. This limitation to systematic reviews rather than original scientific papers was considered appropriate due to the large volume of literature in this area, in-line with recommendations by Smith et al. who recommend the use of review of reviews to allow findings from separate reviews to be synthesised when many systematic reviews exist in a single area (Smith et al., 2011). Reviews published from the data of database conception to April 2020 were included. The last date of search was 5^th^ April 2020. To ensure there were no additional relevant measures, the reviews and reference lists of the reviews were also manually checked. The review was registered on PROSPERO under CRD42018085859.

### Inclusion and Exclusion Criteria

The paper was included if it was a systematic review, reported on adherence measures which included at least one self- or observer-report questionnaire or scale (though the review could include other types of measures including objective measurements of adherence), and a full text English article was available. Reviews which only reported on factors associated with adherence, without including any adherence measure at all (e.g., discussed only adherence factors, or only included satisfaction measures), or reviewed adherence interventions only (i.e., did not include adherence measures), were excluded. Systematic review protocols without published results were also excluded.

### Data Extraction and Analysis

All identified abstracts were screened using the Rayyan tool (Ouzzani et al., 2016) by three researchers (AC, HL, VC) independently, with 100% overlap. Any disagreements were solved by consensus discussion. One researcher (VC) then reviewed and data extracted from the full texts of all identified papers for consistency, with an independent second review and extraction by a second researcher (AC or HL). All papers were therefore reviewed and extracted by at least two researchers independently.

Data extraction was done in two stages. First, data was extracted from the systematic reviews, including: the aim of the review, databases or sources searched in the review, search terms used, questionnaire selection criteria, number of studies included, number of adherence measures used, and names of the adherence measures. Secondly, the original full-text for each measure identified in the reviews was obtained to allow evaluation of what the adherence measure was assessing. Where possible, the full wording of the items in each measure was obtained from the original validation papers or by contacting the author of the measure.

Each measure was then coded according to whether the measure assessed perceptual or practical factors (or both), and whether or not the scale measured adherence (medication-taking) behavior. A measure was considered to measure perceptual factors if it included some reference to necessity beliefs or concerns about treatment, or an individual’s perceptions, attitudes, opinions or emotions towards treatment, or the impact of these perceptions or beliefs on quality of life. A measure was not classified as perceptual if it included only a quantification of the individual’s perceptions or beliefs (e.g., *how often or how much* the patient was concerned about their medication). Practical measures were any questionnaires which included reference to adherence barriers of a non-perceptual nature (i.e., not related to necessity or concerns). Measures were deemed to include a measure of adherence if it captured past or current medication-taking behavior. Questions relating to future medication-taking behavior were not considered to be measures of adherence (as these were considered to be more relevant to intention to take a medication).

Measures were included for final analysis if they were “generic” (i.e., the measure or items within the measure were not related to, or developed for, a specific named condition or medication e.g., blood pressure medication), and included one or more items relating to practical factors. This formed our final inclusion criteria for the adherence measures. Measures could either be self-administered (i.e., self-reported measures) or administered by another person (e.g., a healthcare professional or carer)—i.e., observer-reported measures. The practical items were summarised then analysed thematically by two independent researchers (AC, HL) (Braun and Clarke, 2006). We elected to use the World Health Organisation’s five interacting dimensions that affect adherence as a guiding framework for categorisation of the items (Sabaté, 2003). In line with Braun and Clarke’s approach to thematic analysis, each researcher independently reviewed and familiarized themselves with the measures, generated initial codes, identified common themes, reviewed the themes, then defined and named the themes (Braun and Clarke, 2006). This process was conducted iteratively, with any discrepancies resolved by in-depth discussion and negotiated consensus.

## Results

**Figure 1:**
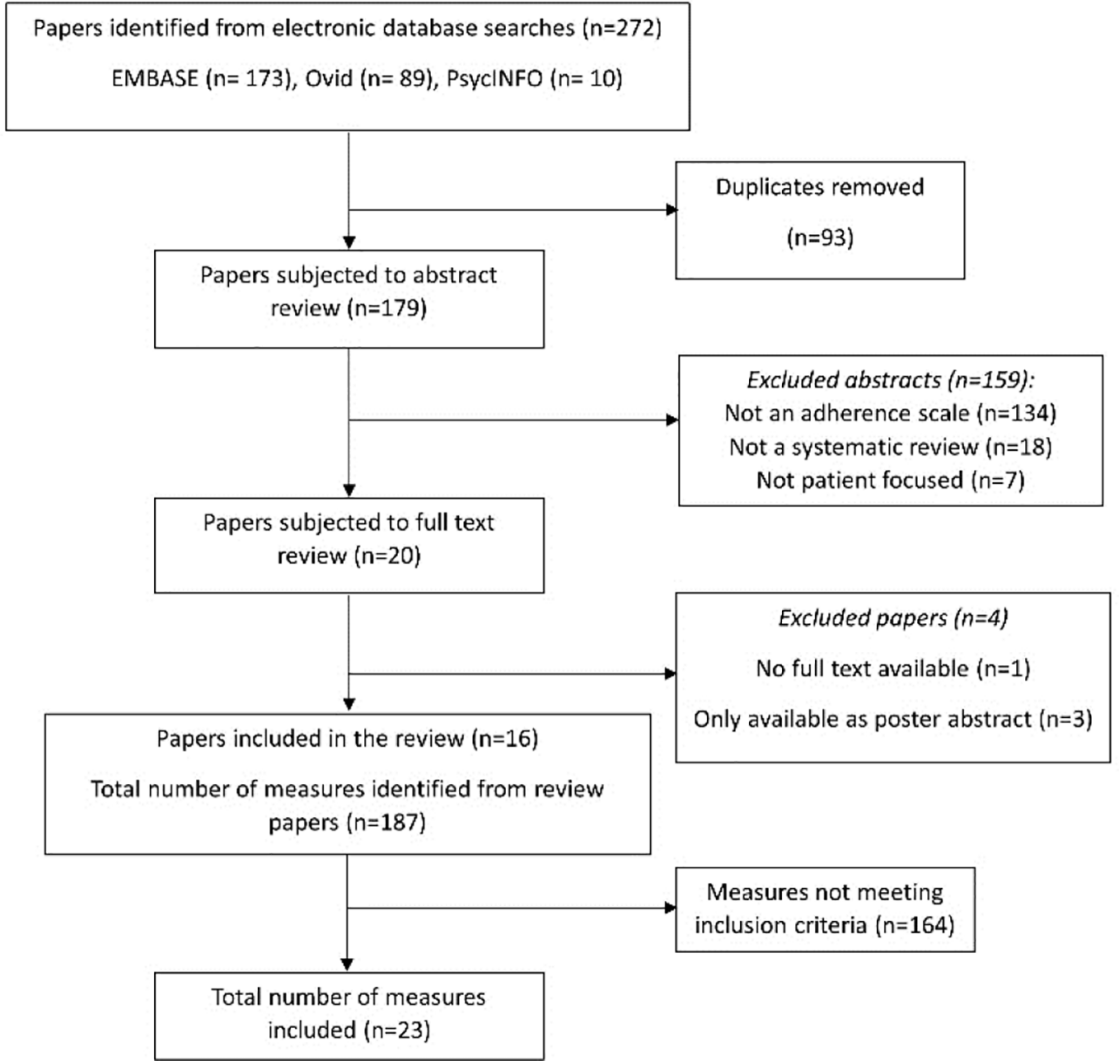
PRISMA flow chart illustrating the selection of studies.

A total of 272 abstracts were initially identified for inclusion, of which 93 were duplicates, leaving 179 for screening. The PRISMA flow diagram which illustrates the study selection process is shown in **Figure 1**. After applying the initial inclusion criteria for the systematic reviews, the full texts of 20 systematic reviews were obtained. Four were excluded after full-text review, leaving 16 systematic reviews for data extraction (Vreeman et al., 2008; Elliott and Marriott, 2009; Vandenbroeck et al., 2011; AlGhurair et al., 2012; Paquin et al., 2013; Remor, 2013; Nguyen et al., 2014; Snyder et al., 2014; Akeroyd et al., 2015; Pérez-Escamilla et al., 2015; Huang et al., 2016; Katusiime et al., 2016; Forbes et al., 2018; Pednekar et al., 2019; Pareja-Martinez et al., 2020; Plevinsky et al., 2020). From these 16 systematic reviews, 187 different adherence measures were extracted and coded. Of these, 23 measures met the final inclusion criteria for the selection of adherence measures, and were included in the thematic analysis.

### Characteristics of Measures

**Table 1** summarizes the characteristics of the included measures. Of the 23 included measures, 3 assessed perceptual barriers and adherence behavior as as well as practical adherence barriers. Nine assessed both perceptual and practical adherence barriers (but not adherence), and 4 evaluated adherence behavior and practical barriers (but not perceptual factors). Perceptual barriers addressed included items relating to concern about long-term effects and side effects of the medicine, and beliefs about the importance or necessity of the medication. Although all the included measures included practical barriers to adherence, only 7 measures solely focused on this area without evaluating perceptual barriers or adherence behavior [TBQ (Tran et al., 2012), DRUGS (Edelberg et al., 1999), MedTAKE (Raehl et al., 2002), MedMaIDE (Orwig et al., 2006), SM task (Isaac and Tamblyn, 1993), MRCI (George et al., 2004), Barriers to treatment maintenance (Cummings et al., 1982)]; these were largely task-based tools which involved a second person observing the patient perform a task (e.g., open a container), or recall information (e.g., state dosing frequency). All measures, with the exception of two measures—the AMBS and PBMS (Simons and Blount, 2007)—were developed in adult populations.

**Table 1:** Characteristics of included measures.

Measure	Number of questions	Administration method	Practical barriers identified (listed by theme number – see **Table 2**)	Adherence measured?	Perceptual barriers measured?
Adherence Attitude Inventory (AAI) (Sarah and Neil, 2002)	34	Self-administered	3	Yes	No
Adherence Estimator (McHorney, 2009)	3	Self-administered	5	No	Yes
Adherence Starts with Knowledge - 12 (ASK-12) (Matza et al., 2009)	12	Self-administered	2, 3, 5, 6	Yes	Yes
Adherence Starts with Knowledge-20 (ASK-20) (Hahn et al., 2008; Matza et al., 2008)	20	Self-administered	1, 2, 3, 4, 5, 6	Yes	Yes
Adherence to Refills and Medications Scale (ARMS) (Kripalani et al., 2009)	14	Interviewer assisted	3, 5, 6	Yes	No
Adolescent Medication Barriers Scale (AMBS) (Simons and Blount, 2007)	17	Self-administered	1, 2, 3, 4, 6, 7	No	Yes
Barriers to treatment maintenance (Cummings et al., 1982)	4 separate measures, total 12 items	Trained interviewers	4, 5, 6	No	No
Beliefs and Behavior Questionnaire (BBQ) (George et al., 2006)	30	Self-administered	4, 5, 6	Yes	Yes
Brief Medication Questionnaire (BMQ) (Svarstad et al., 1999)	9	Research pharmacist	2, 4, 6	Yes	No
Drug Regimen Unassisted Grading Scale (DRUGS) (Edelberg et al., 1999)	4 tasks	Task-based tool	4	No	No
Living with Medicines Questionnaire (LMQ) (Krska et al., 2014; Krska et al., 2017)	60	Self-administered	3, 4, 6	No	Yes
Medication Adherence Reasons Scale (MAR-Scale) (Unni and Farris, 2015)	15	Self-administered	1, 3, 4, 5, 6, 7	No	Yes
Medication Management Instrument for Deﬁciencies in the Elderly (MedMaIDE) (Orwig et al., 2006)	20	Non-medical study staff – questionnaire and task-based	4, 6	No	No
Medication Regimen Complexity Index (MRCI) (George et al., 2004)	65 (over 3 sections)	Researchers	1, 2, 4	No	No
MedTake (Raehl et al., 2002)	4	Task-based tool	2, 4	No	No
Parent Medication Barriers Scale (PMBS) (Simons and Blount, 2007)	16	Completed by the parent/caregiver	1, 2, 3, 7	No	Yes
PROMPT-QoL (Sakthong et al., 2015)	43	Self-administered	2, 4, 6, 7	No	Yes
Simplified Medication Adherence Questionnaire (SMAQ) (Knobel et al., 2002)	6	Healthcare professionals (physician, nurse, pharmacist)	3	Yes	No
SM Task (Isaac and Tamblyn, 1993)	5	Task-based tool	4	No	No
Treatment Burden Questionnaire (TBQ) (Tran et al., 2012)	13	Self-administered	1, 2, 3	No	No
Treatment Satisfaction Questionnaire for Medication abbreviated (TSQM-9) (Bharmal et al., 2009)	9	Self-administered	2, 4	No	Yes
Treatment Satisfaction Questionnaire for Medication V1.4 (TSQM) (Atkinson et al., 2004)	14	Self-administered	2, 4	No	Yes
Treatment Satisfaction with Medicines Questionnaire (SATMED-Q) (Ruiz et al., 2008)	17	Self-administered	1, 2	No	Yes

*This information derives from the main publications that described the original measures; information relating to administration method refers to who administered the measure within the reported study.

The mean ± SD number of items included in the measure was 19 ± 17. Over half of the included measures were self-administered scales (n=13), the remaining 10 were a combination of task-based tools (n=3) and measures administered by study staff or interviewers (n=5), health professionals (n=1), or by the parent/caregiver (n=1). Almost none of the observer-rated scales (with the exception of the parental-completed measure)evaluated perceptual barriers.

### Practical Factors—Themes

The practical barriers within the included adherence measures fell in seven key themes. These are described below, and listed in **Table 2** with examples from each of the measures. These themes were generally spread evenly across all the measures; most measures included items relating to instructions for use (theme 2) or capability (theme 4) with social barriers (theme 7) being captured by only four measures. No measure captured all the practical barriers across the seven themes.

**Table 2:** Practical factors influencing adherence identified from questionnaires.

Theme number	Practical Barrier category	Included factors
*1*	*Formulation*	Taste of tablets Shape of tablets Size of tablets Swallowing difficulties Inconvenience caused by injections (e.g., pain, bleeding, scars)
*2*	*Instructions for use*	Dosing frequency Total number of medicines needed to take (pill/medication burden) Storage of medication (e.g., ease of bringing medicines around/fridge/storage requirements/convenience of carrying the medication during travel or outside work (transport/storage) Restrictions whilst on the medicine (e.g., on food/diet/alcohol/driving) Administration requirements (at time of administration) (e.g., needing to stay upright post administration/taking the medication with specific fluid/dosing at specific times of day) Variable dose pattern (e.g., dose varies/tapering dose) Side effect burden (e.g., such as increased urination limiting activities) Therapeutic drug monitoring requirements (including lab tests/any required doctor visits/self-monitoring requirements (e.g., blood sugars)
*3*	*Issues with remembering*	Busy schedule (e.g., time needed to take medication) Difficulties establishing medication routine
*4*	*Capability—knowledge and skills*	Reading and understanding dispensing labels Difficulties with opening container/packaging Not understanding health provider instructions Being confused or having difficulty identifying what each of medicine does Calculating correct dose Cutting pills to get correct dose Knowing names of medicines Knowing time of administration, how to take, why they are on the medication, amount to take
*5*	*Financial*	Direct: Cost of medication Indirect: Travel fares, monitoring costs to treat your disease/other costs General financial difficulties: Meeting insurance or medication funding criteria
*6*	*Medication supply*	Pharmacy does not have supply Patient has run out of medications Needing to obtain refills or scripts Not having medicine on hand Not knowing where or how to get supply Transport issues to access healthcare or problems with collecting medicines (e.g., transport, parking space, or self-help for the journey)
*7*	*Social environment*	Social influences impeding medication-taking (e.g., reluctance to take medication in front of friends or in a public place) Embarrassment around medication-taking Stigma associated with certain medication (e.g., psychotropic or antiretroviral medicines)

#### 1. Formulation

The formulation theme related to factors around the specific formulation of the medication, such as the size of the oral dosage form (e.g., large tablets) or difficulties associated with injections, which were identified to influence adherence. Six of the included measures had items relating to formulation.

#### 2. Instructions for Use

Many of the measures identified (n=11) included items relating to issues with taking the medication as prescribed, and the burden associated with the medication-taking. This included items ranging from dosing frequency, to medication storage requirements, to specific restrictions with medication administration such as the need to take with food or at certain times of the day. Any items which impacted on daily activities such as an individual’s social life, work, or holidays were included under this category, including indirect medication effects such as the impact of side effects or medication monitoring on daily activities.

#### 3. Issues With Remembering

The third theme relates to issues with remembering or forgetting doses. Ten of the measures included items related to difficulties establishing a medication routine such as forgetting to take medication due to busy schedules or being away on holiday.

#### 4. Capability—Knowledge and Skills

Most measures (n=14) had items which fell into this theme. This relates to an individual’s ability to understand the administration instructions, for example dispensing labels (i.e., relates to health literacy), and to follow the specified instructions (e.g., their ability to open containers, cut pills, or calculate the correct dose). It encompasses an individual’s knowledge and skills which enable them to take the medication as prescribed.

#### 5. Financial

Issues relating to finance or cost were included in a separate theme relating to finance. These included items relating to the direct costs, such as the cost of the medication, or indirect costs relating to medication-taking, such as travelling expenses to obtain the medication supply. Some items related to general finance barriers which are specific to a health system, for example, obtaining health insurance or meeting funding criteria for a medication. Seven of the measures included items on this.

#### 6. Medication Supply

This theme describes adherence barriers which relate to obtaining or accessing medication supplies. It includes issues around the availability of medication and ease of supply of the medication. Eleven of the measures included items in this theme, such as knowing where to obtain medication refills, and being able to get to a pharmacy to obtain ongoing supply.

#### 7. Social Environment

The last theme relates to social influences around medication-taking. It broadly includes any barriers to adherence which arise due to social pressures, such as embarrassment around taking medication in front of other people, and stigma relating to use of medication. Only four of the measures included items relating to this theme, of which two of them related specifically to concerns in adolescents/children about other people noticing them take the medication.

## Discussion

This review of reviews is the first to systematically identify and synthesise the types of practical adherence barriers that are currently captured by self- or observer-reported adherence measures. Recent papers examining factors relating to non-adherence identified practical barriers as key factors influencing adherence (Taylor et al., 2002; Weiser et al., 2003; Martin et al., 2005; Shubber et al., 2016; Jamison et al., 2017). Yet, much of the literature focuses either on the measurement of adherence itself, or on perceptual barriers, or both, with few focusing on the practical issues influencing adherence. Whilst it is important to target both perceptual and practical barriers (Linn et al., 2013), and there is often an overlap between these (Horne et al., 2019), practical barriers represent a good starting point for improving adherence. Practical barriers are generally more easily overcome by simple interventions (e.g., changes to the physical environment or medication regimen) (Horne et al., 2019) and may be useful as an initial adherence intervention. Identifying practical barriers may allow health professionals to make simple interventions to improve adherence first before trialling more resource-intensive interventions to shift perceptual barriers. Additionally, changes in practical barriers can lead to changes in perceptual barriers; patients may become more motivated to take their treatment if the medication regimen was made simpler and easier to take. Conversely, many of the practical barriers identified from our review can be overcome if patients are highly motivated—for example, patients are more likely to follow complicated regimens and defy practical barriers if they believe their treatment is necessary. However, shifting an individual’s beliefs about their treatment often requires a more intensive approach that can only be delivered by individuals who have received extra training, e.g., delivery of a health psychology-based intervention, compared to addressing practical barriers, which health professionals may find easier to deliver (Chapman et al., 2015).

This review identified 23 adherence measures which assessed practical barriers to adherence. There were few self- or observer-reported measures identified that addressed only practical barriers, with most measures consisting of only a few items addressing practical barriers. Where measures did focus primarily on practical factors, these were administered in the form of task-based tools, where an observer scored tasks performed by the individual, rather than self-report measures.

Our review builds on previous literature by undertaking a review of systematic reviews of self- and observer-reported adherence measures to ensure that all practical barriers previously identified in adherence measures were identified. It extends the work conducted by Nguyen et al., who conducted a review of primary literature around self-report adherence measures, but did not focus on practical adherence barriers (Nguyen et al., 2014). The findings of this review provide key information for intervention development and the design of future adherence measures, particularly ones that target practical barriers. Previous literature have highlighted the importance of tailoring adherence interventions, with a recent meta-analysis reporting “tailoring” as the most effective behavior change technique to consider when designing an adherence intervention (Kassavou and Sutton, 2017). Valid and reliable measures for tailoring interventions according to individual barriers exist (Horne et al., 1999; Linn et al., 2013), yet from our review, there was no single measure that specifically focused on practical factors nor captured all the barriers represented across the seven themes. From our review, separating perceptual and practical factors is important for intervention development, as perceptual and practical barriers require different types of intervention, with different resource intensity. The themes from this review provide a foundation for the development of a measure that can identify the practical adherence barriers faced by an individual. Used together with measures which evaluate perceptual barriers, such as the Beliefs about Medicines Questionnaire, such a measure would facilitate the development of a tailored adherence intervention that addresses both perceptual and practical barriers—an approach in-line with current guidance from NICE (Horne et al., 2005; Nunes et al., 2009).

The measures identified were broadly consistent with each other, with all statements relating to practical factors falling into one of the seven overarching themes that identified in this review. The themes that were identified aligned well with previous literature including the World Health Organisations’ five dimensions that affect adherence, which we used as a guiding framework for analysis (Sabaté, 2003). Most measures contained items that measured capability, such as opening medication bottles, and whether patients understood the proper administration of their medicines, with items assessing whether patients knew how and when to take the medication. Although self-report is a convenient method of collecting information, a key limitation is that an individual’s *perceived* ability may not reflect the actual ability of the individual to administer the medication; in this case observer-reported (e.g., task-based) measures would be more accurate. We found that in our current review, over half of the measures were self-reported. There is scope for the development of more observer-reported measures, or for measures to adopt a mix of self- and observer-report within the questionnaire, to increase the accuracy of assessment of practical barriers. Of note, most of these observer-reported measures also did not evaluate perceptual barriers, which makes sense as perceptual barriers are much more likely to be accurately identified by the individual themselves, whereas practical barriers may be more accurately identified by an observer. Observer-reported measures may thus play a more predominant role in identifying practical barriers; whilst self-reported measures may be more suited for perceptual barriers. Further research on observer-reported measures is warranted. Additionally, only two measures focused specifically on adherence barriers in children and adolescents. This represents a gap for further research in identify the barriers that face young people, and how these compare to adults and to the barriers that parents/caregivers perceive their children face. Furthermore, identification of barriers does not necessarily translate into actual adherence; identifying the specific barriers is only the first step towards improving adherence. It is also not known whether certain types of practical barriers (e.g., financial) may have a greater influence on adherence; future work to examine the relationships between the different types of practical barriers reported in our review and how these impact on adherence is needed.

The themes relating to the other items were generally spread evenly across the measures, however, social influences such as embarrassment were identified only in four measures; of these two were social influences considered in the context of child and adolescent adherence. However, as the papers that reported on the development of the adherence measures had little information on where the measure items were derived from, the frequency of occurrence of these items do not necessarily reflect the frequency with which these practical barriers are experienced by individuals. Furthermore, a possible reason for the lack of inclusion of social influences in adherence measures may reflect the difficulty associated with quantifying this construct, compared to more concrete task-based barriers such as cost or obtaining medication supplies.

### Strengths and Limitations

We chose to use a review of systematic reviews approach to formulate an understanding of the existing literature and to explore the extent to which practical barriers have been included in current adherence measures. The approach proved valuable for identifying and synthesising the practical factors currently evaluated across multiple adherence measures. However, because the search only captured systematic reviews, any new measures developed which have not been yet been included in prior systematic reviews will not have been picked up in this review of reviews. Our review was also limited to only “generic” scales to maximize the generalizability of our findings across different conditions and disease states. However, with generalisability comes the loss of specificity, and practical barriers which are unique to a particular treatment or condition will not have been included. We also chose not to apply quality screening criteria for the systematic reviews, such as AMSTAR 2 (Shea et al., 2017), as the reviews in this case were used to identify measures used in the literature rather than synthesising outcomes or findings.

## Conclusion

This review has identified and categorised practical factors that can lead to unintentional non-adherence. Many of the themes relating to practical adherence barriers reflected situations which are out of the patients’ direct control, yet could be easily addressed if identified. There is currently no self- or observer-reported measure that assesses these barriers independently of other factors, which could lead to many of these practical factors being overlooked. The findings from this review provide a useful foundation on which to develop a measure of practical barriers to adherence, and to inform intervention design.

## Author Contributions

AC was involved in the study design and conception, study search, article screening, data extraction, data analysis and interpretation, and manuscript write-up. VC was involved in the study design, study search, article screening, data extraction, data analysis and interpretation, and manuscript write-up. HL was involved in the study search, article screening, data extraction, data analysis and interpretation, and manuscript write-up. RH was involved in the study design, data interpretation, and manuscript write-up and review.

## Funding

The authors declare that this study received funding support from Spoonful of Sugar Ltd., a UCL-Business spin-out company. The funder was not involved in the study design, collection, analysis, interpretation of data, the writing of this article, or the decision to submit it for publication.

## Conflict of Interest

AC reports consultancy fees from Janssen-Cilag, and Spoonful of Sugar Ltd, and speaker fees from Novartis, outside the submitted work. RH reports fees from Medical Innovation Academic Consortium (CASMI), AbbVie, Amgen, Biogen, Idec, Gilead Sciences, GlaxoSmithKline, Janssen, Pfizer, Roche, Shire Pharmaceuticals, MSD, Astellas, AstraZeneca, DRSU, Novartis, Universit€atsklinikum Hamburg-Eppendorf, and Teva Pharmaceuticals, and is the Founder and Director of Spoonful of Sugar Ltd., outside the submitted work. VC and HL are employees of Spoonful of Sugar Ltd.

## Appendix 1

Search terms:

1. Measure*.mp. or Patient Reported Outcome Measures/
2. Inventor*.mp.
3. Scale*.mp. or Visual Analog Scale/
4. “Surveys and Questionnaires”/or Questionnaire*.mp.
5. Self Report/
6. instrument.mp. or Psychometrics/
7. 1 or 2 or 3 or 4 or 5 or 6
8. Adherence*.mp. or Medication Adherence/
9. Patient Compliance/or Compliance*.mp.
10. 8 or 9
11. barrier*.mp.
12. factor*.mp.
13. Health Knowledge, Attitudes, Practice/
14. belief*.mp. [mp=title, abstract, original title, name of substance word, subject heading word, keyword heading word, protocol supplementary concept word, rare disease supplementary concept word, unique identifier, synonyms]
15. Percep*.mp.
16. practical*.mp.
17. forget*.mp.
18. enable*.mp.
19. facilitat*.mp.
20. 11 or 12 or 13 or 14 or 15 or 16 or 17 or 18 or 19
21. valid*.mp. or Psychometrics/
22. 7 and 10 and 20 and 21
23. (Measure* or Patient Reported Outcome Measures or Inventor* or (Scale* or Visual Analog Scale) or (“Surveys and Questionnaires” or Questionnaire*) or Self Report or (instrument or Psychometrics)).ti. or (Measure* or Patient Reported Outcome Measures or Inventor* or (Scale* or Visual Analog Scale) or (“Surveys and Questionnaires” or Questionnaire*) or Self Report or (instrument or Psychometrics)).ab.
24. (Adherence* or Medication Adherence or (Patient Compliance or Compliance*)).ti. or (Adherence* or Medication Adherence or (Patient Compliance or Compliance*)).ab.
25. (valid* or Psychometrics).ti. or (valid* or Psychometrics).ab.
26. 20 and 23 and 24 and 25
27. systematic review.mp.
28. 26 and 27
